# Monitoring and evaluation of sport-based HIV/AIDS awareness programmes: Strengthening outcome indicators

**DOI:** 10.1080/17290376.2016.1266506

**Published:** 2016-12-20

**Authors:** Elma Nelisiwe Maleka

**Affiliations:** ^a^ PhD student at the Interdisciplinary Centre for Sports Science and Development, University of the Western Cape, Bellville, South Africa; ^b^ Postdoctoral research fellow at the School of Public Health, University of the Western Cape, Bellville, South Africa

**Keywords:** HIV/AIDS, indicator, non-governmental organisations, outcome, performance assessment, sport-for-development, VIH/SIDA, indicateur, organisations non gouvernementales, résultats, évaluation de la performance, sport au service du développement

## Abstract

There are number of Non-Governmental Organisations (NGOs) in South Africa that use sport as a tool to respond to Human Immunodeficiency Virus/Acquired Immunodeficiency Syndrome (HIV/AIDS), however, little is reported about the outcomes and impact of these programmes. The aim of this study is to contribute to a generic monitoring and evaluation framework by improving the options for the use of outcome indicators of sport-based HIV/AIDS awareness programmes of selected NGOs in South Africa. A qualitative method study was carried out with seven employees of five selected NGOs that integrate sport to deliver HIV/AIDS programmes in South Africa. The study further involved six specialists/experts involved in the field of HIV/AIDS and an official from Sport Recreation South Africa (SRSA). Multiple data collection instruments including desktop review, narrative systematic review, document analysis, one-on-one interviews and focus group interview were used to collect information on outcomes and indicators for sport-based HIV/AIDS awareness programmes. The information was classified according to the determinants of HIV/AIDS. The overall findings revealed that the sport-based HIV/AIDS awareness programmes of five selected NGOs examined in this study focus on similar HIV prevention messages within the key priorities highlighted in the current National Strategic Plan for HIV/AIDS, STIs and TB of South Africa. However, monitoring and evaluating outcomes of sport-based HIV/AIDS programmes of the selected NGOs remains a challenge. A need exists for the improvement of the outcome statements and indicators for their sport-based HIV/AIDS awareness programmes. This study proposed a total of 51 generic outcome indicators focusing on measuring change in the knowledge of HIV/AIDS and change in attitude and intention towards HIV risk behaviours. In addition, this study further proposed a total of eight generic outcome indicators to measure predictors of HIV risk behaviour. The selected NGOs can adapt the proposed generic outcomes and indicators based on the settings of their programmes. A collaborative approach by all stakeholders is required, from international organisations, funders, governments, NGOs and communities to strengthening monitoring and evaluation of sport-based HIV/AIDS awareness programmes including other development programmes. This will assist the NGOs that use sport for development to be able to reflect accurately the information about their HIV/AIDS activities and also be able to contribute to on-going monitoring activities at a national and global level as well as to the Sustainable Development Goals.

## Résumé

Plusieurs organisations non gouvernementales (ONG) en Afrique du Sud utilisent le sport comme un outil de réponse au Virus d'Immunodéficience Humaine/Syndrome d'Immunodéficience Acquise (VIH/SIDA). Cependant peu d’études ont été faites sur les résultats et l'impact de ces programmes. Le but de cette étude est de contribuer à un cadre de suivi et évaluation générique en améliorant les options pour l'utilisation d'indicateurs de résultats dans les programmes de sensibilisation au VIH / Sida basés sur le sport, d'ONG Sud-Africaines sélectionnées. Une étude utilisant une méthode qualitative a été réalisée avec sept employés de cinq ONG sélectionnées qui intègrent le sport dans la mise en œuvre de programmes VIH/SIDA en Afrique du Sud. L'étude a aussi porté sur six spécialistes/experts impliqués dans le domaine du VIH/SIDA et un responsable de Sport Recreation South Africa (SRSA). Des instruments multiples de collecte de données, incluant une revue documentaire, une revue narrative systématique, l'analyse de documents, des entrevues individuels et des groupes de discussion ont été utilisés pour recueillir des informations sur les résultats et les indicateurs des programmes de sensibilisation au VIH/SIDA basés sur le sport . L'information a été classée en fonction des déterminants du VIH/SIDA. Les résultats généraux révèlent que les programmes de sensibilisation au VIH/SIDA basés sur le sport de cinq ONG sélectionnées et examinées dans cette étude portent sur des messages de prévention du VIH similaires aux priorités qui sont soulignées dans l’actuel Plan stratégique national pour le VIH/SIDA, les IST et la tuberculose de l’Afrique du Sud. Toutefois, le suivi et l'évaluation des résultats des programmes VIH/SIDA basés sur le sport des ONG sélectionnées restent un défi. Il existe un besoin d'amélioration des énoncés des résultats et des indicateurs de leurs programmes de sensibilisation au du VIH/SIDA axés sur le sport. Cette étude propose un total de 51 indicateurs de résultats génériques, mettant l'accent sur la mesure du changement des connaissances sur le VIH/SIDA, et le changement d'attitude et d'intention envers des comportements à risque liés au VIH. En outre, cette étude propose un total de huit indicateurs de résultats génériques pour mesurer les prédicteurs de comportements à risque liés au VIH. Les ONG sélectionnées peuvent adapter les résultats et les indicateurs génériques proposés en fonction des paramètres de leurs programmes. Une approche concertée de tous les acteurs est nécessaire, incluant les organisations internationales, les bailleurs de fonds, les gouvernements, les ONG et les communautés pour pouvoir renforcer le suivi et l'évaluation des programmes de sensibilisation au VIH/SIDA basés sur le sport, y compris d'autres programmes de développement. Cela aidera les ONG qui utilisent le sport comme outil de développement à pouvoir refléter avec précision les informations sur leurs activités liées au VIH/SIDA et également à pouvoir contribuer aux activités de suivi en cours au niveau national et mondial, ainsi qu’aux objectifs de développement durable (ODD).

## 1. Introduction

With regard to any new development initiatives, there is a big focus on Human Immunodeficiency Virus/Acquired Immunodeficiency Syndrome (HIV/AIDS) prevention among communities across the world. Monitoring and evaluation of such programmes is critical and should move beyond outputs to the assessment of achieved anticipated outcomes. According to UNAIDS (2014 2015), assessing the progress made against the targets using indicators is critical to assist governments and Non-Governmental Organisations (NGOs) to better understand their responses to the HIV pandemic. Sport is widely known for its health benefits through direct participation in sport itself and as platform for communication, education and social mobilisation (SDP IWG, 2008a). Sport has recently been highlighted as a tool in contributing to the achievement of Sustainable Development Goal 3: ‘Good Health and Wellbeing’ which involves responding to HIV/AIDS (UNOSDP 2015). Despite growing support of sport-based HIV/AIDS interventions, there is still a gap in monitoring and evaluation of such initiatives (Kicking AIDS Out, 2010). There are limited specific studies conducted to provide an insight on the use of outcome indicators for the monitoring and evaluation of sport-based HIV/AIDS awareness programmes. Some of these studies tend to focus on too many aspects of monitoring and evaluation at the same time which led to limited emphasis on outcome indicators. Moreover, these studies relied on one method of inquiry. For instance, Coalter (2008) relied mainly on the multiple case study approach by involving four projects that use sport as a tool for development. On the other hand, Kaufman, Spencer, and Ross (2013) used only a systematic review.

The aim of this study is to contribute to a generic monitoring and evaluation framework by improving the options for the use of outcome indicators of sport-based HIV/AIDS awareness programmes of selected NGOs in South Africa. This will be achieved through the use of different sources to collect information including different data collection instruments to identify and develop indicators that are applicable to a generic monitoring and evaluation framework regarding the outcomes of sport-based HIV/AIDS awareness programmes of selected NGOs in South Africa. Developing relevant indicators is essential to assess progress, measure achievements and reflect the changes connected to objectives and outcomes of any programmes aimed at development (Kusek & Rist, 2004; Rabie, 2014). The National Strategic Plan for HIV, STIs and TB of South Africa (NSP-SA) 2012–2016, recommends that all initiatives aimed at responding to HIV/AIDS should focus on well-formulated objectives and targets to be able to demonstrate their contribution to the goals of the NSP (NSP-SA, 2012). International organisations like UN, WHO, USAID, UNGDF and CDC have developed guidelines on indicators that can be adapted and used according to a country’s setting to monitor and evaluate progress of HIV/AIDS programmes (UNAIDS, 2009).

### 1.1. The role of sport as a tool to respond to HIV/AIDS

Worldwide, both developed and developing countries support sport-based interventions to meet various social needs and to achieve the Millennium Development Goals (MDGs) (Kicking AIDS Out, 2010; Sport and Development Organisation, 2014). The findings of the interviews conducted by SDP IWG (2008b) among 34 countries reveal that all countries agreed that sport can be used as a tool to address various social needs. According to SDP IWG (2008b) out of 34, 18 countries identified a connection between their Sport for Development and Peace policies and the achievement of the MDGs 2, 3 and 6, MDG 2: to achieve universal primary education, MDG 3: to promote gender equality and empower women and MDG 6: to combat HIV and AIDS, malaria, and other diseases (SDP IWG, 2008b). In the recently developed Sustainable Development Goals, the role of sport is highlighted as an important enabler for Sustainable Development (UNOSPD, 2015).

Numerous toolkits and curricula on how to use sport activities to respond effectively to HIV/AIDS have been developed and are mostly used by NGOs. Organisations such as Grassroot Soccer, Kicking AIDS Out, International Olympic Committee (IOC) and UNAIDS in partnership with several health organisations have played a significant role to ensure that sport is used to explore issues on HIV/AIDS. Grassroot Soccer has curriculum in use to provide a series of activities to deliver HIV/AIDS messages and life skills particularly among youth (Khan & Hendrin, 2010). According to USAID (2010b), these activities are interactive and aimed at creating and reinforcing resilience among youth. According to USAID (2010b), activities such as ‘Find the Ball’ (where two teams each pass a ball labelled ‘HIV’ behind their backs and each participant has to identify who is holding the ball), is used to teach participants that it is not possible to tell if someone is infected with HIV by just looking at them (USAID, 2010b). The activity further promotes HIV testing and also discourages stigma and discrimination towards people living with HIV/AIDS (PLWHA) (USAID, 2010b). Another example is ‘Risk field’ which explores risky behaviours through dribbling a ball around cones that represent different risky behaviours (Khan & Hendrin, 2010). There are other health messages linked to football skills that are in use. Fuller, Junge, Dorasami, DeCelles, and Dvorak (2011) give examples as follows, ‘passing a ball representing respect girls and women, heading meaning protect yourself from HIV/AIDS and dribbling meaning avoid drugs and alcohol’.

It can be argued that sport can be used to address various aspects of HIV/AIDS. Therefore there is need to understand theories that underpin sport-based HIV/AIDS programmes. According to Mwaanga (2010) sport can be used for moral support. For instance, participation in sport programmes can contribute in developing life skills such as self-esteem and self-confidence that can motivate self-care to promote healthy lifestyle behaviours (IOC & UNAIDS, 2010; Mwaanga, 2010; SDP IWG, 2008a).

The Magic Bus organisation integrated sport to develop a curriculum on sexual and reproductive health (SRH) including HIV/AIDS and life skills to empower youth in India (Magic Bus, 2014). According to Pandey (2013), the review of the Magic Bus’s SRH content found the curriculum sufficient to be able to promote HIV/AIDS awareness. The curriculum was also considered as adequate by Pandey (2013) to provide young people with life skills such as negotiating and thinking skills to be able to resist peer pressure. As part of the review, Pandey (2013) also conducted focus group interviews with youth who participated in the SRH programme. According to Pandey (2013) the findings indicate that there was a positive change in their perception regarding condom use, gender stereotypes, gender-based discrimination and violence.

Similarly, Kicking AIDS Out (2010), Mwaanga (2010) and Tobisch and Preti (2010) argue that sport can be used as a ‘hook’ to attract young people. This can be done through the training of coaches and also through using sport ambassadors to promote HIV/AIDS prevention messages (Tobisch & Preti, 2010). For example the organisation SCORE trains coaches and leaders to be able to use sport to drive development including HIV/AIDS prevention messages (SCORE, 2014). Furthermore a different evaluation study conducted by Clark, Friedrich, Ndlovu, Neilands, and McFarland (2006) demonstrates that it was possible to use professional soccer stars to deliver messages aimed at reducing risks of HIV/AIDS in Zimbabwe. The findings of Maro, Roberts, and Sørensen (2009) reflected that using trained peer coaches through sport can be an effective tool to minimise risks of HIV infection among youth in sub-Saharan Africa. In South Africa, a football-based health promotion programme implemented by trained coaches indicates a significant increase in health knowledge among youth participants compared to a control group (Fuller *et al*. 2010).

Sport can also be used as a means to foster empowerment and to improve health for PLWHA (Mwaanga, 2010). According to Tobisch and Preti (2010) sport-based programmes provide suitable space to change attitude of young people towards HIV/AIDS, HIV testing including reducing stigma towards PLWHA. It is therefore critical that these theories are explored and taken into consideration when designing sport-based HIV/AIDS programmes (Mwaanga, 2010).

Khan and Hendrin (2010) conducted an evaluation among 16 NGOs using football-related activities to respond to HIV/AIDS in Africa. The findings reveal that all 16 NGOs are using a variety of approaches that incorporate these theories to conduct their HIV/AIDS prevention programmes. South Africa is among other countries that recognise a need to use sport to meet health challenges related to HIV/AIDS (SDP IWG, 2008b). The commitment of Sport Recreation South Africa (SRSA) to respond to HIV/AIDS is clearly highlighted in their strategic plan and policy (SRSA, 2012; SRSASP, 2012). NGOs such as Whizzkids United ‘On the Ball’, Grassroot Soccer and AMANDLA EduFootball, are amongst other NGOs worldwide who have developed a culture of routine data collection, documenting and publicising their evaluation results for their life skills and HIV/AIDS awareness programmes for targeted youth.

Whizzkids United is a non-profit organisation that uses football as a teaching tool to promote HIV/AIDS awareness among youth in Durban, KwaZulu Natal. These include teaching life skills that aimed at helping youth to adopt healthy behaviours (Farrar & Math, 2010, 2012). In 2010, the organisation conducted an evaluation of Whizzkids United ‘On the Ball’ intervention. The findings of the evaluation revealed a significant increase in 19 of the 30 behavioural predictors, of which 6 out of the 19 predictors were linked to prevention of HIV/AIDS (Farrar & Math, 2010). Again in 2012, Whizzkids United’s final evaluation also showed that there was a statistically significant improvement in HIV/AIDS knowledge, attitude, gender norms, self-efficacy and future orientation of youth who participated in their programmes (Farrar & Math, 2012). It was concluded that the impact of the programme was mostly found in HIV/AIDS knowledge, attitude and self-efficacy (Farrar & Math, 2012). Another example is the evaluation of AMANDLA EduFootball which according to AMANDLA EduFootball (2011) shows an improvement in the youth behavioural pattern of fair play and of life skills. The fair play concept is used during games to discourage high levels of violence conducted within football (AMANDLA EduFootball, 2011).

The researcher has studied these three evaluations reports in the context of community development work at a grassroot level. These organisations, Whizzkids United and AMANDLA EduFootball have shown that through ongoing monitoring and evaluation there is a potential for sport to be used as an engine in advancing development programmes. However, very few sport-based organisation conduct monitoring and evaluation in South Africa. In addition, Keim (2006) argues that there is too little recognition of how sport is used at community level for development purposes.

The literature reviewed in this section demonstrates that it is possible to use sport as a medium of education to respond to HIV/AIDS. The studies also reveal that sport-based HIV/AIDS awareness programmes address the same risky behaviours that put young people at high risks of HIV infection. However, responding effectively to HIV/AIDS requires rigorous planning, management, monitoring and evaluation of all forms of HIV/AIDS prevention programmes (Coates, Ritcher, & Caceres, 2008; Gayle, 2008). This should involve comprehensive assessment before, during and after implementation to be able to conduct rigorous evaluation to measure knowledge, attitude and behaviour change of participants (Kaufman *et al*. 2012; Mercy Corps, 2007; Mwaanga, 2010).

Authors like Kruse (2006), Coalter (2008, 2010) and Kaufman et al. (2012, 2013) recommend that evaluation of sport-based HIV/AIDS programmes should include clear indicators assessing progress towards achieving sporting and non-sporting outcomes. A review by Kaufman et al. (2013) found that NGOs use different indicators to measure behavioural outcomes of their sport-based HIV/AIDS prevention programmes and thus make it difficult to determine effectiveness. Therefore, a specific need exists to identify and develop appropriate indicators to evaluate outcomes of sport-based HIV/AIDS awareness programmes. These indicators should be developed in such a way that can be easily adapted to the need of any NGO providing sport-based HIV/AIDS awareness programme. In the South African context, this will create a platform to establish standardised reporting tool that can be used by NGOs to showcase their work in a credible manner. In addition, it can serve to inform policy makers and other health sectors about the effectiveness of sport-based HIV/AIDS awareness programmes as well as identifying areas that require improvement.

### 1.2. Overview of monitoring and evaluation

It can be argued that monitoring and evaluation are theoretical concepts that are closely related to each other and they both provide important evaluative information (Forss, Befani, & Kruse, 2012). There is an increase in demand for a shift from traditional monitoring and evaluation which mainly focuses on assessing inputs, outputs and implementations process. In the context of development, the focus should further include other factors that contribute in achieving outcomes and impact (IEG, 2012; Kusek & Rist, 2004; Puvimanasinghe, Gill, & Beck, 2007; UNDP, 2002). It is very important that government and NGOs clearly differentiate between outcomes, outputs and activities in order to be able to clarify contribution of each towards the long-term objective of any given programme or project (OECD, 2011).

#### 1.2.1. Purpose of monitoring and evaluation

Monitoring and evaluation is undertaken for different purposes and is normally used to measure progress and performance towards programme or project objectives (Görgens & Kusek, 2009; Kusek & Rist, 2004; Mackay, 2007). This involves a systematic collection and analysis of data to provide information about what works, what does not work and the reasons why (Astbury, 2013; DBE & MIET Africa, 2010; Görgens & Kusek, 2009; Mackay, 2007; Morra Imas & Rist, 2009; Puvimanasinghe *et al*. 2007; UNAIDS, 2010). The information should be used to identify weaknesses and provide recommendations to improve programmes or projects (Kusek & Rist, 2004; UNAIDS, 2010). Monitoring and evaluation is used to demonstrate outcomes and impact of programmes or projects, to identify potential programmes or projects and also to explore unintended results (Berriet-Solliec, Labarthe, & Laurent, 2014; Kusek & Rist, 2004; Rabie & Cloete, 2011). Monitoring and evaluation is used for financial accountability within governments and organisations (Kusek & Rist, 2004). It is important to have a clear understanding of the monitoring and evaluation and its different components.

#### 1.2.2. Theoretical perspective on developing indicators

International and national efforts to implement programmes aimed at development require governments and NGOs to demonstrate results achieved (Chan *et al*. 2010). In this context, the word ‘results’ specifically focuses on monitoring outcomes and evaluating impact to determine what difference did the programme make (Mallett, Talley, & Harris, 2011; OECD, 2010). This requires a consultative process among stakeholders to agree on the programme’s goals, objectives, inputs, activities, outputs, outcomes and impacts alongside measurement indicators (Group on Earth Observations, 2008; IEG, 2012; National Treasury, 2007; W.K. Kellogg Foundation, 2004). A result-based monitoring and evaluation system serves as an effective tool that can be used by governments and NGOs to track progress and make adjustments to any given programme (Kusek & Rist, 2004; Mackay, 2007, 2008). This creates an opportunity to be able to follow a right direction that will support and lead to the achievement of the desired outcomes (Mackay, 2008; Morra Imas & Rist, 2009). Outcomes are the short- and medium-term effects of an intervention’s outputs and the changes that we desire to achieve as a results of the programme (National Treasury, 2007; OECD, 2010). Once the outcomes are clearly articulated, the next key step is to develop and select performance indicators to measure whether the desired outcomes are being achieved or not (Kusek & Rist, 2004). Church and Rogers (2006) claim that ‘in any evaluation, indicators act as signals of change occurred during the programme’. Indicators can either be quantitative or qualitative (Kusek & Rist, 2004; UNDP, 2009; USAID;, 2010a). The process of selecting indicators should be a participatory process within the organisation, stakeholders including experts in that particular field (Coll-Serrano, Carrasco-Arroy, Blasco-Blasco, & Vila-Lladosa, 2012; Kusek & Rist, 2004; Rabie, 2011; Rogers, Chappelle, Wall, & Barron-Simpson, 2011; Simister, 2009; Taplin, Clark, Collins, & Colby, 2013; UNDP, 2002, 2009; USAID, 2010a). This will assist in identifying what can be monitored and evaluated given financial resources and human capacity (UNDP, 2009). Frameworks such as performance framework and outcome logic model should be considered to assist in developing indicators that are relevant to outcomes to be measured (IEG, 2012; Kusek & Rist, 2004; Mallett *et al*. 2011; Rabie, 2011; W.K. Kellogg Foundation, 2004). There are several steps that should be considered when selecting indicators. These steps are presented below according to the approaches of Görgens and Kusek (2009) USAID (2010a) and Rabie (2011).

Step 1: Develop a participatory process for identifying performance indicators

Step 2: Clarify the results statement, identify what needs to be measured

Step 3: Develop a list of possible indicators for your results through brainstorming and research

Step 4: Assess each possible indicator

Step 5: Select the best indicators

Step 6: Data indicator protocols

Step 7: Collect baseline data

Step 8: Refine indicators and protocols and finalise you selection

These approaches guided the process of identifying and developing indicators that are applicable to a generic monitoring and evaluation framework regarding the outcomes of sport-based HIV/AIDS awareness programmes of selected NGOs in South Africa.

## 2. Methodology

A qualitative method study was carried out in South Africa with seven employees of five selected NGOs that integrate sport to deliver HIV/AIDS programmes. The study further involved six specialists/experts involved in the field of HIV/AIDS and one official from SRSA. Three phases of data collection using multiple data collection instruments were followed to contribute to a generic monitoring and evaluation framework by improving the options for the use of outcome indicators of sport-based HIV/AIDS awareness programmes of selected NGOs in South Africa. In this light, phase 1 included a desktop review of existing indicators in use to monitor or evaluate outcomes of HIV/AIDS awareness programmes and a narrative systematic review of outcome indicators currently in use for sport-based HIV/AIDS awareness programmes. A narrative synthesis approach was used to analyse and summarise data from the reviews. In addition, an approach of USAID (2011) on outcome and effective integration model was adapted and used to analyse data from the narrative systematic review.

Phase 2 was divided into three sections namely, 2(a): document analysis, 2(b): one-on-one interviews and 2(c): focus group interview. In phase 2(a), document analysis of the documents such as annual reports, M&E and project plans of selected NGOs was conducted. In phase 2(b), a purposive sampling was used to select seven employees suitable for one-on-one interviews. A focus group with same employees that were interviewed during phase 2(b) was conducted in phase 2(c). Document analysis, one-on-one interviews and a focus group were used to comprehend what indicators are in use to monitor and evaluate outcomes of sport-based HIV/AIDS awareness programmes of the selected NGOs. Cross-case analysis using thematic approach was used to analyse data from document analysis and one-on-one interviews (Creswell, Hanson, Plano Clark, & Morales, 2007; Yin, 2014). Thematic analysis was used to analyse data from the focus group (Creswell, 2009).

Phase 3 included the use of a convenience sampling to select six specialists/experts involved in the field of HIV/AIDS to explore what indicators can be used to monitor outcomes of sport-based awareness HIV/AIDS programmes. In addition, a purposive sampling was used to select one official from SRSA who is involved in monitoring and evaluation. Schedule one-on-one interviews were used to collect data from the specialists/experts including an official from SRSA. Thematic approach was used to analyse data (Creswell, 2009). Prior to schedule one-one interviews, the draft outcome indicator framework was sent to six specialists/experts and an official from SRSA by email. The participating specialists/experts including an official from SRSA were requested to provide comments, suggestions, recommendations on the value and relevance of the proposed indicators as well as suggestions for refinement of the generic anticipated outcomes and indicators. In addition, to recommend possible or alternative outcome indicators to improve the quality of proposed outcome indicator framework. They were also requested to provide the comments in a form of writing after two weeks. Six specialists/experts including an official from SRSA responded and sent their comments via email.

The researcher conducted the reviews, document analysis and all the interviews with employees of selected NGOs, experts/specialists and an official from SRSA. A focus group interview with employees of selected NGOs was conducted by a Post-Doctoral Fellow and a researcher as an observer. During phase 2(a) and 2(b), face**-**to**-**face one-on-one interviews and focus group interview were conducted. In phase 3, interviews with three specialists/experts and an official from SRSA were face-face. In addition, interviews with other three specialists/experts were conducted using telephone. All the interviews were prepared and transcribed by the researcher.

Ethics clearance was obtained from the University of the Western Cape (UWC). The UWC information sheet was utilised that clearly outlines the purpose, risks and benefits of the study, requirements procedures to keep confidentiality and a letter asking permission to conduct this study. Permission to conduct, record interviews and have access to documents was obtained from the employees of the selected NGOs, specialists/experts and an official from SRSA.

The following strategies according to Creswell (2009) were used to ensure trustworthiness and accuracy of the findings.

Triangulation of data - document analysis and focus group interview were used to complement the information gained from one-on-one interviews and to ensure accuracy and gain clarity.

Member checking - the researcher served as a check throughout the data collection and analysis processes and was involved in all stages of data collection and analysis.

## 3. Results and discussions

The results will be presented and discussed simultaneously according to the phases used to collect data. Results and discussions from the reviews are presented first, followed by document analysis, one-on-one interviews and focus group. The next section presents results and discussions from the interviews with specialists/experts and an official from SRSA. The results and discussions from all phases with particular focus on anticipated outcomes and indicators will be integrated and used to contribute to a generic outcome indicator framework for sport-based HIV/AIDS programmes of selected NGOs in South Africa.

### 3.1. Desktop review

Indicators to measure outcomes focusing on sexual and behavioural determinants of HIV/AIDS were identified from the desktop review. The sexual and behavioural determinants of HIV/AIDS are listed according to NSP-SA (2012), Shisana et al. (2009, 2014) and UNAIDS (2014, 2015) as prevention knowledge and risk perceived, early sexual debut, multiple concurrent sexual partners, condom use, HIV Counselling and Testing (HCT), transactional sex and intergenerational sex. In addition, indicators to measure outcomes focusing on measuring the prevalence of intimate partner violence against women and stigma associated with HIV/AIDS were also identified. The identification of indicators relied mostly on the use of the guidelines from United Nations Millennium Development Goals (2008), DBE (2012), USAID (2013), Office of Disease Prevention and Health (2014) and UNAIDS (2014, 2015). Furthermore, evaluation reports of NGOs such as Vision and Soul City Institute Health and Development were used to identify indicators (Keating, Meekers, & Adewuyi, 2006; Soul City Institute Health and Development, 2010). These indicators will be considered when identifying the proposed outcome indicators for sport-based HIV/AIDS awareness programmes of the selected NGOs examined in this study.

### 3.2. Narrative systematic review

A systematic review following a narrative synthesis was used to ascertain the scope of existing indicators used in monitoring and evaluating the outcomes of sport-based HIV/AIDS awareness programmes. Popay et al. (2006) and Rodgers et al. (2009) define a narrative synthesis as an approach to a systematic review that relies primarily on the use of words and text to explain and summarise the findings of the synthesis. In other words, it is identification of qualitative information. This was achieved through the review of some of the available published and unpublished evaluation studies of sport-based HIV/AIDS awareness programmes.

#### 3.2.1. Selection of studies for inclusion

##### 3.2.1.1. Types of participants

This review included sport-based HIV/AIDS awareness interventions in all countries targeting youth and adults. For the purpose of this study youth refers to people younger than 18 years, youth refers to people between the age group of 18–35 years. Adults refer to people who are above the age of 35 years.

##### 3.2.1.2. Types of interventions

Intervention had to use sport as a tool to promote HIV/AIDS awareness. This included educational interventions as well as interventions aimed at reducing sexual and other behavioural risks, stigma related to HIV/AIDS, increase uptake of health services that contribute to a reduction of HIV/AIDS transmission such as HCT, sexual transmission infections (STIs) and awareness and access to antiretroviral treatment.

##### 3.2.1.3. Types of study designs

This review included studies that meet the following inclusion criteria:

Published and unpublished studies

Published studies refer to peer-reviewed articles published in both local and international journals. Unpublished studies refer to evaluation reports of both local and international sport-based HIV/AIDS awareness programmes.

- Published and unpublished studies that evaluate sport-based HIV/AIDS programmes to raise HIV/AIDS awareness and reduce HIV/AIDS transmission among youth and adults in any country.
- Published and unpublished studies issued in the last 10 years, i.e. between 1 January 2004 and 31 December 2014.
- Published and unpublished studies either using qualitative or quantitative measures employing randomised or non-randomised trials, cross-sectional, case-control studies comparing intervention and control group or pre-post comparison.

##### 3.2.1.4. Language

In this evaluation, only published and unpublished studies written in English were included.

##### 3.2.1.5. Types of outcome measures

Studies were included if they reported one or more of the key HIV/AIDS awareness outcome such as biological, sexual behaviour, condom use, HIV/AIDS, STI and pregnancy incidence, knowledge of HIV/AIDS, attitude and self-efficacy, gender norms, stigma, service uptake/referral to HCT and antiretroviral therapy (ARV).

#### 3.2.2. Search strategy

A review used a two-way search strategy to identify published and unpublished studies. A search to identify peer-reviewed articles was conducted using electronic databases like EBSCO, SCOPUS, MEDLINE, EMBASE, Google Scholar, hand searches of the reference lists of key journals and the university library. Furthermore, cross reference lists of selected articles, reports, websites related to indicators to measure outcomes of sport-based HIV/AIDS awareness programmes were reviewed. Google was also used to search relevant unpublished studies and evaluation reports of sport-based HIV/AIDS programmes in order to ascertain which indicators are in use. Search criteria included keywords such as HIV, AIDS, sport-based HIV/AIDS programme, sport-based HIV/AIDS awareness/prevention programme, effectiveness, monitoring, evaluation, outcomes, results and indicators.

#### 3.2.3. Data extraction and management

For all studies and programme reports consulted, inclusion and exclusion criteria were extracted either from the abstract or from the full article or report. Selected articles and reports were carefully examined by the researcher and the information about objectives, outcomes and indicators of sport-based HIV/AIDS programmes was extracted using the logical models of Kusek and Rist (2004) and USAID (2011). All the process of conducting a systematic review was performed and verified by the researcher in consultation with the supervisors.

#### 3.2.4. Data extraction and assessment tool

Data were extracted through identification of qualitative information, or indicators in this instance. The qualitative information was sorted thematically according to the stated objectives, reported outcomes and description of findings and measurable indicators from the included studies. Use was also made of thematic frequency analysis as well as theme-based cross-cutting analysis to identify causal relationships.

#### 3.2.5. Data extraction and presentation of included studies

A total of 14 evaluation studies on sport-based HIV/AIDS awareness programmes were included and conducted for the period between 2006 and 2012. Of the 14 studies, 7 were published in 4 peer journals, 6 studies were evaluation reports of sport-based HIV/AIDS programmes while the last one was an unpublished Master of Public Health-thesis. Studies were conducted in various countries: four in South Africa, two in Zimbabwe, one in Kenya, Mauritius, Dominican Republic, Tanzania, Burkina Faso, Liberia, Southern Sudan and Zambia. The study designs included four quasi-experimental studies, eight pre/post cohort studies, one cross-sectional study and one parallel cohort study. The target population for included studies ranged in the age group 9–30 years.

The information was first classified according to the objectives, reported outcomes and description of findings from included studies. A total of 17 objectives and 66 outcomes were identified from the included studies. The next step was an analysis of the reported outcomes and findings discussed using an outcome integration model adapted from USAID (2011) and these are presented in Section 3.2.7. Of the 66 outcomes, a total of 23 core outcomes were aligned. Furthermore, a total of 27 indicators used to measure these outcomes were identified. These indicators are relevant for measuring outcomes which are focusing on the following: change in HIV risk behaviours, change in predictors of HIV risk behaviours, change in attitude, belief and intention towards HIV risk behaviours and change in the knowledge of HIV/AIDS. These indicators will be considered when identifying and proposed outcome indicators for sport-based HIV/AIDS awareness programmes of the selected NGOs examined in this study.

#### 3.2.6. Key outcomes from included studies

The approaches of USAID (2011) on outcome and effective integration model were adapted and used to quantify the outcomes across all 14 included studies. This involved classification of outcomes according to the following categories: health or biological, knowledge of HIV/AIDS, communication about HIV/AIDS, sexual and behavioural transmission of HIV/AIDS, gender norms within the context of HIV/AIDS, gender norms concerning sports, process outcomes: awareness to HIV/AIDS services, and stigma. Studies reporting on each outcome were counted and discussed. Studies associated with improved outcomes were classified as positive. Furthermore, studies with no statistically significant difference in the outcomes and also with joint positive and negative effects were counted and classified as mixed or with no effect. Studies that showed worse outcomes were classified as negative and studies with inconclusive findings were classified as inconclusive (USAID, 2011).

#### 3.2.7. Discussions: key outcomes from included studies

##### 3.2.7.1. Health or biological outcomes

No studies reported on change in health or biological outcomes. However, one ultimate goal of a study by Farrar and Math (2010) was to enable HIV-positive youth to manage infection and maintain their health. Three health outcomes namely: reduced HIV prevalence, increased CD 4 count and reduced viral load were mentioned but not measured due to limited resources. According to Farrar and Math (2010), in an ideal situation such kind of evaluation would require a longitudinal randomised controlled study comprising of an intervention group and a control group. This would further require a repeatedly comparison of HIV status of this two group including the assessment of CD4 counts and viral loads of those who are HIV infected (Farrar & Math, 2010).

##### 3.2.7.2. Knowledge of HIV/AIDS outcomes

- Improved knowledge of HIV/AIDS: Twelve studies reported on change in the knowledge of HIV/AIDS outcomes. Of the 12 studies, 9 reported improvement in the knowledge of HIV/AIDS while 2 studies showed no effect and one study by Carter (2006) had inconclusive findings. Fuller et al. (2010) reported no dramatic change in the level of knowledge related to HIV/AIDS in any of the groups from pre to post and three months after post. Another study by Peacock-Villada, DeCelles, and Banda (2006) reported a slight difference in both correct responses of boys and girls from baseline to post-test.
- Increased communication about knowledge of HIV/AIDS: Three studies reported on communication about HIV/AIDS and they all showed a positive effect.

##### 3.7.2.3. Sexual and behavioural of transmission of HIV/AIDS outcomes

Thirteen outcomes were reported under predictors of sexual and behavioural risks of HIV/AIDS. They were either reported as attitude, belief, experience, intention or subjective norms towards that particular outcome. The most commonly reported sexual and behavioural outcome was on abstinence, condom use and concurrent partners respectively.

- Delayed sexual experience: Two studies reported on sexual experience and neither of them had positive effect. A study by Delva et al. (2010) showed no significant difference in the fraction of respondents reported to have ever had sex in the intervention group and in the control group, while study by Carter (2006) reported inconclusive findings.
- Positive attitude towards delayed sexual debut: Of the two studies reported on attitude towards delayed sexual debut, one had positive effect. Another study by Delva et al. (2010) showed no effect on attitude towards delaying sexual debut in the intervention group as well in the control group.
- Increased perceived risk-avoiding behaviours: Three studies reported on perceived risk-avoiding behaviours and two showed a positive effect and one did not had any effect. Delva et al. (2010) showed no significant differences on reported perceived risk-avoiding behaviours between the intervention group and the control group.
- Increased condom use: Two studies reported direct experience on condom use. Both showed positive results. In addition, there were six additional outcomes related to condom use and are discussed below.
- Positive intention towards condom use and being faithful to your partner: One study reported by Delva et al. (2010) showed no significant differences on reported behavioural intention towards condom use and remaining faithful to a partner between the intervention group and the control one.
- Positive attitude/belief towards condom use: Three studies reported on attitude/belief towards condom use. All three studies that reported on attitude/belief towards condom use showed positive results.
- Positive attitude towards consistent condom use: One study reported on attitude towards consistent condom use and showed a positive effect.
- Increased perceived behavioural control/self-efficacy in condom use: Two studies reported on perceived behavioural control/self-efficacy in condom use and both showed positive effect.
- Increased percentage of respondents reporting having one or more condoms with them: One study reported on having one or more condoms with them and there was an improved outcome.
- Increased awareness of where to access condoms (not having difficulty finding a place to buy condoms): One study reported on access to condoms and had a positive effect.
- Positive attitude towards abstinence: Seven studies reported on attitude, beliefs and subjective norms towards abstinence, five showed a positive effect and two had a mix effect. Peacock-Villada et al. (2006) was recorded as having mix effect because the findings reported a slight difference in correct responses of boys and no difference in girls from baseline to post-test. Fuller et al. (2010) was also recorded as having mix effect because the findings showed negative effect in the intervention group from pre-post and then positive effect after three months.
- Decreased concurrent sexual partners: Three studies reported on history, attitude and subjective norms towards concurrent sexual partners and two had a positive effect and one showed no effect. Delva et al. (2010) reported no significant differences in the percentage of respondents reporting to have ever had concurrent relationships either for respondents in MYSA and for respondents in the control group.
- Increased belief in exclusive sexual partner: One study reported on attitude and subjective norms to exclusive sexual partner and showed a positive effect.
- Increased subjective norms on virginity and responsibility: One study by Delva et al. (2010) showed no significant differences on reported subjective norms to virginity and responsibility between the intervention group and the control group. As a result the study was recorded as having no effect.
- Positive attitude towards uptake of HIV testing: Two studies reported on willingness to get tested for HIV. One study showed a positive effect and one had inconclusive results. Carter (2006) reported inconclusive findings on willingness to get tested for HIV.

##### 3.2.7.4. Gender norms with the context of HIV/AIDS outcomes

Three outcomes were reported under gender norms with the context of HIV/AIDS. The reported outcomes are on sexual violence, equality in relationship and respect for girls. The most commonly reported gender norms outcome was on respect for girls. One study reported on sexual violence and one on equality in relationship.

- Improved knowledge that sexual violence increases risks of HIV infection: One study reported on sexual violence and showed improvement in positive responses on sexual violence.
- Increased belief in equality in relationship: One study reported on norms, beliefs and attitude towards equality in relationship and showed positive effect.
- Positive attitude towards respect for girls: Three studies reported on norms, belief and attitude towards respect for girls. Of the three studies, two reported positive effects and one showed mix effect. A study by Fuller et al. (2010) was recorded as having mix effect because the findings showed negative effect in the intervention group from pre-post and then positive effect after three months.

##### 3.2.7.5. Increased equitable gender norms concerning sports outcomes

Four studies reported on equitable gender norms concerning sports and all had a positive effect.

##### 3.2.7.6. Awareness of health services outcomes

One study reported on awareness of HIV/AIDS prevention services in community and showed a positive effect.

##### 3.2.7.7. Stigma and discrimination related to HIV/AIDS outcomes

Reduced stigma: Seven studies reported on stigma (attitude towards PLWHA). Six studies reported positive effect and a study by Carter (2006) reported inconclusive findings.

The findings of the outcome and effective integration model show that there were no negative outcomes or effect reported, however they were few that showed mix or no effect. The findings on the knowledge of HIV/AIDS indicate that the participants were knowledgeable before the intervention. There were no significant differences in the correct responses of the intervention group and control one from baseline to post in two studies (Fuller *et al*. 2010; Peacock-Villada *et al*. 2006). As it is suggested by Fuller et al. (2010), this calls for the development and selection of HIV/AIDS knowledge questions that are age-appropriate. In addition, the questions should be relevant to the specific target audience. The NGOs can make use of the guideline on 100 HIV/AIDS knowledge questions and answers developed by Gallant (2007). They can also follow the guidelines of UNAIDS (2013, 2014, 2015) and Shisana et al. (2014).

Overall, the findings of the narrative systematic review indicate that the sport-based HIV/AIDS awareness programmes have similar objectives that are also in line with the commitments and targets of the 2011 UN Political Declaration on HIV/AIDS. The sport-based HIV/AIDS awareness programmes are addressing risky sexual behaviours that place individual at high risk of contracting HIV/AIDS. The sport-based HIV/AIDS awareness programmes of included study are targeting young people as they are being identified in UNAIDS (2013) as the most group at risk to HIV infection. It is important to acknowledge that it is very difficult to measure sexual, behavioural and biological outcomes. As pointed out by Farrar and Math (2010) evaluation of sexual, behavioural and biological outcomes would require longitudinal studies which are often too costly. Conducting a longitudinal study requires collection of data from same sample of participants on multiple occasions to track changes over time, as well as relating them to variables that might provide explanation on why those particular changes occur (Lynn, University, 2015). Therefore, the findings of this systematic review showed that included studies rely mainly on measuring change in the knowledge of HIV/AIDS, attitude and belief towards HIV risks behaviours including self-reported predictors of HIV risk behaviours. Similar to findings of Kaufman et al. (2013) there were no studies that measured health or biological outcomes. However, Farrar and Math (2010) identified three health outcomes namely: HIV prevalence, CD4 counts, viral load and can be measured if there are adequate financial and human resources.

Kaufman et al. (2013) reported that one of the challenges observed in the evaluation of sport-based HIV/AIDS programmes is the heterogeneity of indicators used to measure behaviour outcomes. Similar findings were observed in this review, however, the approaches of USAID (2011) enable this study to classify the outcomes into relevant categories and also to align and select core outcomes from the included studies. This made it possible to identify 27 indicators that are currently in use by included studies to monitor and evaluate outcomes of sport-based HIV/AIDS awareness programmes. The findings of the narrative systematic review can assist the NGOs that use sport as a tool to respond to HIV/AIDS to improve the measurement of their anticipated outcomes. The NGOs can adapt and use these indicators according to their settings and their sport-based HIV/AIDS awareness programmes.

Although the narrative systematic review did not assess how rigorous were the research methods and designs used in the studies. It can be concluded that the review demonstrated that sport-based HIV/AIDS awareness programmes do have a meaningful contribution in raising awareness about HIV risk behaviours including uptake of health services. The challenge is the question on how can they be further assisted in order to allow an opportunity to monitor and evaluate health or biological outcomes including change in risk behaviours. This can be an ongoing argument that involving governments, funders, NGOs, researchers, evaluators and all relevant stakeholders who have an interest in improving the evaluation of sport-based HIV/AIDS awareness programmes. The findings further suggested that evaluating outcomes of sport-based HIV/AIDS awareness programmes of included studies require the use of appropriate research design in order to track changes over time.

### 3.3. Document analysis and one-on-one interviews

The results revealed that the HIV prevention messages, objectives and anticipated outcomes of the sport-based HIV/AIDS awareness programmes of the selected NGOs are in line with the focal areas for change identified in the 2011 Political Declaration on HIV/AIDS and the National Strategic Plan for HIV/AIDS, STIs and TB of South Africa (NSP-SA, 2012; UNAIDS, 2013, 2014, 2015). All selected NGOs have a set of outputs alongside indicators and are disaggregated by gender and age. Furthermore, all sport-based HIV/AIDS awareness programmes of all NGOs have set of anticipated outcomes. The majority of the anticipated outcomes appeared to be similar amongst selected NGOs. However, only one NGO has a set of indicators to measure anticipated outcomes of sport-based HIV/AIDS awareness programme. The anticipated outcomes of the selected NGOs focus on the following themes: reduce sexual transmission of HIV/AIDS, promote uptake of ARV, care and support and support services for people living with HIV, eliminating gender inequalities, eliminating stigma and discrimination and promoting HCT and Voluntary Medical Male Circumcision (VMMC). Other outcomes focus on awareness regarding health, psychosocial and other support services. One outcome focus on using mass HIV/AIDS sessions during sport events to attract participants to come and participate in small group HIV/AIDS sessions offered at centres/sites. Although the NGOs have anticipated outcomes which are in line with the key determinants of HIV/AIDS, others have insufficient information about some changes that the NGOs desired to achieve. For instance, some anticipated outcomes do not include the target population while others are not clear on what is to be measured. A need exists for the improvement of the outcome statements of the selected NGOs. This will allow an opportunity to identify performance indicators (Kusek & Rist, 2004).

As reported by Kaufman et al. (2013), one of the challenges experienced in the evaluation of sport-based HIV/AIDS awareness programmes is the heterogeneity of indicators used to measure anticipated outcome. A need exists for the identification and development of a variety of generic outcome indicators to strengthen monitoring and evaluation of the outcomes of sport-based HIVAIDS awareness programmes in South Africa. This study indicated that measuring the anticipated outcomes of the selected NGOs requires the selection of indicators that rely mostly on predictors of sexual, behaviour and social determinants of HIV/AIDS. The changes on the anticipated outcomes would rely on self-reported HIV risk behaviours among participants, including change in attitude, belief and intention towards HIV risk behaviours. Similar findings were also observed on evaluation studies of sport-based HIV/AIDS awareness programmes of the included studies of the narrative systematic review conducted in phase 1 of this study (Carter, 2006; Clark *et al*. 2006; Delva *et al*. 2010; Farrar & Math, 2010, 2012; Fuller *et al*. 2010, 2011; Kaufman *et al*. 2012; Luppe, 2010; Maro *et al*. 2009; Mercy Corps, 2007; Peacock-Villada *et al*. 2006).

### 3.4. Discussions from focus group interview

The results from a focus group interview with employees of selected NGOs indicated a need to classify the anticipated outcomes according to the determinants of HIV/AIDS. In addition, the employees identified specific risk sexual behaviours that they were addressing during their sport-based HIV/AIDS awareness programmes. The findings also provided clarity regarding the target audience. All employees of selected NGOs who participated in the focus group interview agreed that participants refer to any participants reached with sport-based HIV/AIDS awareness programme/intervention that integrates HIV/AIDS awareness and life skills. These are offered during/at sport events, tournaments and trainings as well as at hubs/centres/sites. The sport-based HIV/AIDS awareness programmes of selected NGOs operate for a year depending on the availability of funding. Majority of their target population are in school-learners. Therefore, it was concluded that measuring their anticipated outcomes requires measurement of short-term change relying on self-reported predictors of HIV risk behaviours, change in knowledge, attitude and intention towards HIV risk behaviours. Similarly to the views of Farrar and Math (2012), the employees of selected NGOs were concerned that it might be challenging to measure directly the contribution of sport-based HIV/AIDS awareness programmes of selected NGOs in reducing the rate of HIV infection. The selected NGOs do not have capacity and resources to ensure that measuring HIV incidence is done in accordance with the biomedical ethics and standards. Furthermore, they do not use control groups either.

### 3.5. Discussions from specialists/experts and an official from SRSA

Overall, the majority of the specialists/experts and an official from SRSA felt that the proposed outcomes were relevant to HIV/AIDS. However they highlighted concerns about the feasibility of measuring change in some HIV risk behaviours including predictors of HIV risk behaviours. They felt that some anticipated outcomes and proposed indicators were possible to measure at a country/national level rather than at an NGO level considering financial resources required and time frames. Some of the concerns of the specialists/experts and an official from SRSA are similar to the ones emerged from the focus group interview with employees of selected NGOs. For instance measuring change in HIV risk behaviours appeared to be a common concern.

#### 3.5.1. Comments from the specialists/experts and an official from SRSA

Measuring sexual and behaviour outcomes is often challenging considering the implications of research design, sample size, causality, time frame and diffusion of knowledge due to other variables.

It is difficult to measure but the most relevant indicator is HIV incidence within a target population.

Measuring sexual and behaviour change outcomes is challenging as it requires the use of longitudinal study which are often too costly to be conducted at NGO level.

It can be practical to measure change in HIV knowledge, change in attitude and intention towards HIV risk behaviours … therefore a need exist to rephrase/redefine these anticipated outcomes and indicators while keeping them relevant to the wishes of the selected NGOs.

It is critical for NGOs to ensure the integrity, accuracy and reliability of the output results/data since they are the foundation of achieving the programme’s desired outcomes.

The selected NGOs should ensure that their sport-based HIV/AIDS awareness programmes are age appropriate in terms of messages aimed at raising awareness about HIV risk behaviours … furthermore, indicators must be adapted and be relevant to different age group levels.

### 3.6. Proposed generic outcome indicators

The aim of this study is to contribute to a generic monitoring and evaluation framework by improving the options for the use of outcome indicators of sport-based HIV/AIDS awareness programmes of selected NGOs in South Africa. The proposed generic outcome indicator framework of this study is based on the findings obtained from different sources using multiple data collection instruments. The proposed generic outcome indicators were identified compiled and/or developed based on the key HIV messages, objectives and anticipated outcomes of sport-based HIV/AIDS awareness programmes of selected NGOs examined in this study. In addition, they were based on the desktop review of existing indicators in use to monitor or evaluate outcomes of HIV/AIDS awareness programmes. Furthermore, they were based on the findings from the narrative systematic review of outcome indicators currently in use for sport-based HIV/AIDS awareness programmes. The recommendations of the specialists/experts and an official from SRSA were also considered. These findings were used to contribute to a generic outcome indicator framework adapted from approach of Rabie (2011). This involved developing a logic model that depicts the relationship between the objectives, anticipated outcomes along with a variety of possible indicators for the measurement of various sport-based HIV/AIDS awareness programmes of selected NGOs in South Africa. The ‘CREAM’ concept of selecting good indicators as defined by Kusek and Rist (2004) referring to clear, relevant, economic, adequate and monitorable was considered to prioritise the usefulness of the proposed indicators to the sport-based HIV/AIDS awareness programmes of selected NGOs.

#### 3.6.1. Description of the proposed outcome indicator framework

The objectives, HIV messages and anticipated outcomes of selected NGOs examined in this study were considered and classified into categories related to the determinants of HIV/AIDS. The generic anticipated outcomes associated with each category were listed under each objective, and then a list of proposed outcome indicators was compiled. Two proposed outcome indicator frameworks were developed. The first framework focuses on sexual, behavioural, structural and biological determinants of HIV/AIDS and is presented in Tables 1–9. This framework consists of objectives, anticipated outcomes and indicators focusing on measuring change in the knowledge of HIV/AIDS, change in attitude and intention towards HIV risk behaviours and condom use. In addition, objectives, anticipated outcomes and indicators focusing on HCT, gender norms, HIV treatment, care and support, VMMC, use of health services, stigma and discrimination. In addition, the first framework also presents objectives, anticipated outcomes and indicators related to positive behaviour and attitude as a result of sport as a tool for social change and are presented in Tables 10 and 11. The second framework is presented in Tables 12 and 13 and consists of the outcome indicators that can be used to measure change in predictors of HIV risk behaviours. In addition, they can be used to measure the actual change in HIV risk behaviours.

**Table 1.** Proposed outcome indicators for knowledge of HIV/AIDS.

Objective: To improve knowledge of HIV/AIDS			
Generic anticipated outcomes	Generic outcome indicators	Sources	Notes
Improved knowledge of HIV/AIDS amongst participants	Percentage of participants who identify correct modes and dispels myths about how HIV/AIDS is transmitted	Grassroot Soccer (2014)	Data to be collected through surveys using HIV knowledge questions Pre & post for intervention group If possible have a control group and do pre & post Indicators should be disaggregated by gender and age
	Percentage of participants who correctly identify ways of preventing sexual transmission of HIV/AIDS and who reject major misconceptions about HIV/AIDS	Carter (2006) Peacock-Villada et al. (2006) Mercy Corps (2007) United Nations MDG (2008) Maro et al. (2009) Fuller et al. (2010, 2011) Farrar and Math (2010, 2012) Luppe (2010) DBE (2012) Kaufman et al. (2012) USAID (2013) UNAIDS (2013, 2014, 2015)

**Table 2.** Proposed outcome indicators for HIV risk behaviours.

Objective: To raise awareness of HIV risk behaviours			
Generic anticipated outcomes	Generic outcome indicators	Sources	Notes
Improved knowledge of benefits associated with delaying sexual intercourse	Percentage of participants who identify benefits associated with delaying sexual intercourse	This study	Data to be collected through surveys using HIV risk behaviours questions Pre & post for intervention group If possible have a control group and do pre & post Indicators should be disaggregated by gender and age
Positive attitude towards delaying sexual intercourse	Percentage of participants reporting positive attitude towards delaying sexual intercourse		
Positive intention to delay sexual intercourse	Percentage of participants reporting to have intention to delay sexual intercourse	Delva et al. (2010) Farrar and Math (2010)	
Improved knowledge of risks associated with having multiple concurrent sexual partners	Percentage of participants who identify risks associated with having multiple concurrent sexual partners	Grassroot Soccer (2014)	
Improved knowledge of risks associated with engaging in transactional sex (provision of sex in exchange for material things, for example, money, airtime, clothing, lunch etc.	Percentage of participants who identify risks associated with engaging in transactional sex	This study	
Improved knowledge of risks associated with age disparate sex/cross-generational sex	Percentage of participants who identify risks associated with having non-marital sex with a partner 10 years older or more than themselves		
	Percentage of adolescents participants who identify risks associated with dating a partner 5 years older than themselves		
	Percentage of participants who identify risks associated with having older sexual partners		
Improved knowledge of risks associated with alcohol and drugs misuse	Percentage of participants who identify risks associated with having sex under the influence of alcohol and drugs misuse		
	Percentage of participants who identify risks associated with having a relationship with a partner who misuses alcohol and drugs

**Table 3.** Proposed outcome indicators for condom use.

Objective: To raise awareness and access to condoms (male and female condoms)			
Generic anticipated outcomes	Generic outcome indicators	Sources	Notes
Improved knowledge of benefits associated with condom use	Percentage of participants who identify how to correctly use a condom and what benefits it provides	Grassroot Soccer (2014)	Data to be collected through surveys using HIV risk behaviours questions associated with condom use Pre & post for intervention group If possible have a control group and do pre & post Indicators should be disaggregated by gender and age
Improved knowledge of benefits associated with consistent condom use	Percentage of participants believing that consistent condom use reduces risks of HIV	Keating et al. (2006)	
Improved knowledge of benefits associated with consistent condom use	Percentage of participants believing that consistent condom use reduces risks of HIV infection, STIs and unwanted pregnancy	This study	
Positive attitude towards condom use	Percentage of participants who feel that a partner is justified in refusing unsafe sex or proposing condom use if they know their partner has STI		
Positive intention to use condom	Percentage of participants reporting intention to use condom at their first sexual intercourse, of those reporting to be not sexual active	Clark et al. (2006) Mercy Corps, (2007) Maro et al. (2009)	
Increased perceived self-efficacy to negotiate condom use with a partner	Percentage of participants reporting perceived self-efficacy to negotiate condom use with a partner	This study	
	Percentage of participants reporting perceived efficacy to refuse unprotected sex	Farrar and Math (2010) Maro et al. (2009)	
Increased awareness about where to access condoms	Percentage of participants reporting to know where to access condoms	Delva et al. (2010)	
	Percentage of participants reporting having easy access to condoms	This study	
	Percentage of participants reporting to be not ashamed to collect or buy condoms

**Table 4.** Proposed generic outcome indicators for HCT.

Objective: To promote uptake of HCT			
Generic anticipated outcomes	Generic outcome indicators	Sources	Notes
Improved knowledge of benefits associated with taking an HIV test	Percentage of participants who identify benefits associated with taking an HIV test	This study	Data to be collected through surveys using questions associated with uptake of HCT Pre & post for intervention group If possible have a control group and do pre & post Indicators should be disaggregated by gender and age
Positive attitude towards uptake of HCT	Percentage of participants reporting willing to go for an HIV test	Carter (2006) Farrar and Math (2010) Grassroot Soccer (2014)	
Increased uptake of HCT services	Percentage of participants graduated from sport-based HIV/AIDS awareness programme tested for HIV and receive/know their result	Grassroot Soccer (2014)	Data to be collected from the Department of Health (DOH) HCT forms that are normally used by testing NGOs, clinics and other testing centres A short surveys can be used during testing campaigns/events asking if a person have ever tested for HIV. This will assist to determine percentage of those who tested for HIV for the first time The DOH HCT forms has a question asking if someone is getting tested for the first time Indicators should be disaggregated by gender and age The HCT data can be compared from the current and the previous years (and targets could be set)
	Percentage of participants graduated from sport-based HIV/AIDS awareness programme first ever tested for HIV	This study	
	Percentage of participants tested for HIV at sport events and receive/know their result		
	Percentage of participants tested for HIV at sport events first ever tested for HIV at sport events

**Table 5.** Proposed generic outcome indicators for gender norms.

Objective: To raise awareness about gender norms that increase risk of HIV infection			
Generic anticipated outcomes	Generic outcome indicators	Sources	Notes
Reduction in gender norms that increase risk of HIV infections	Percentage of participants express gender equity-primarily in regard to gender-based violence	Grassroot Soccer (2014)	Data to be collected through surveys using gender norms as related to HIV/AIDS questions Pre & post for intervention group If possible have a control group and do pre & post Indicators should be disaggregated by gender and age
	Percentage of participants reporting to be against sexual violence	Farrar and Math (2010)	
	Percentage of participants reporting to believe in equality in a relationship		
	Percentage of participants reporting to believe that girls should be respected	Fuller et al. (2010, 2011)	
	Percentage of participants reporting that boys should protect and not harm girls		
	Percentage of participants reporting to believe that football is for boys only	Fuller et al. (2011)	
	Percentage of participants reporting to believe that rugby is for boys only	This study

**Table 6.** Proposed generic outcome indicators for HIV treatment, care and support.

Objective: To promote uptake of HIV treatment, care and support Eligible individuals: participants who test HIV positive through sport-based HIV/AIDS awareness programmes			
Generic anticipated outcomes	Generic outcome indicators	Sources	Notes
Increased percentage of participants referred: HIV care and support services	Percentage of participants who test HIV positive and referred for a tertiary test, clinic enrolment, CD4 counts and ART. Every participant who tests HIV positive should receive one of the service referrals	Grassroot Soccer (2014)	Data to be collected through registers as well as one-one interviews with HIV positive participants Confidentiality should be kept at all stages of communication Indicators should be disaggregated by gender and age
Improved knowledge of benefits associated with taking ARV/HIV treatment	Percentage of HIV positive participants who identify benefits associated with taking ARV/HIV treatment	This study	
Improved knowledge of benefits associated with adhering to ARV/HIV treatment	Percentage of HIV-positive participants who identify benefits associated with adhering to ARV/HIV treatment		
Increased follow-up with HIV positive participants	Percentage of HIV positive participants receive follow-up from the NGOs	Grassroot Soccer (2014)

**Table 7.** Proposed outcome indicators for VMMC.

Objective: To promote uptake of VMMC Eligible individuals: MCUTS 1 and 11 trial participants: This topic should be touched on in all sport-based HIV/AIDS awareness curricular, but the referrals should be explicitly offered only in MCUTS			
Generic anticipated outcomes	Generic outcome indicators	Sources	Notes
Improved knowledge of benefits associated with VMMC	Percentage of male participants who identify the benefits of VMMC and where this service is offered	Grassroot Soccer (2014)	Data to be collected through surveys Pre & post for intervention group If possible have a control group and do pre & post
Increased percentage of male participants referred: VMMC	Percentage of male participants who access information, request, and receive counselling, and are referred for VMMC procedures	Grassroot Soccer (2014)	Data to be collected through registers as well as one-one interviews with male participants Confidentiality should be kept at all stages of communication
	Percentage of male participants reporting to have uptake circumcision after being motivated at the intervention or referred	This study

**Table 8.** Proposed outcome indicators for the use of health services.

Objective: To promote the use of health services Eligible individuals: Any participants reached with sport-based HIV/AIDS awareness intervention			
Generic anticipated outcomes	Generic outcome indicators	Sources	Notes
Increased awareness about health services	Percentage of participants reporting to be aware of any of the following services in their communities: services: HCT, ART, VMMC, PMTCT, STI, antenatal care, post exposure prophylaxis, TB, family planning services and services that deals with gender-based violence	Clark et al. (2006) Grassroot Soccer (2014)	Data to be collected through surveys Pre & post for intervention group If possible have a control group and do pre & post Indicators should be disaggregated by gender and age
	Percentage of participants who have asked coaches/coordinators for referral to HCT, ART, VMMC, PMTCT, STI, antenatal care, post exposure prophylaxis, TB, family planning services and services that deals with gender-based violence	Grassroot Soccer (2014)	Data to be collected through registers as well as one-one interviews with HIV positive participants Confidentiality should be kept at all stages of communication Indicators should be disaggregated by gender and age
	Percentage of participants that have actually been linked to the referred services	This study

**Table 9.** Proposed generic outcome indicators for stigma and discrimination.

Objective 1: To reduce HIV stigma and discrimination towards PLWHA			
Generic anticipated outcomes	Generic outcome indicators	Sources	Notes
Reduced HIV stigma and discriminatory attitude towards PLWHA	Percentage of participants who report accepting attitude towards PLWHA	Carter (2006) Clark et al. (2006) Peacock-Villada et al. (2006) Mercy Corps (2007) DBE (2012) USAID (2013) UNAIDS (2013, 2014, 2015)	Data to be collected through surveys using questions related to HIV stigma and discrimination Pre & post for intervention group If possible have a control group and do pre & post Indicators should be disaggregated by gender and age
	Percentage of participants who are willing to talk, care for, and identify with someone who has HIV/AIDS		
Positive intention to communicate about HIV/AIDS with peers and family	Percentage of participants reported intention to communicate with someone outside of a programme about HIV/AIDS	Clark et al. (2006) Grassroot Soccer (2014)	
Increased percentage of HIV positive participants who report to feel comfortable to disclose HIV status	Percentage of HIV positive participants who express a positive ability to feel comfortable to disclose their HIV status to their sexual partners and/or any person they trust	This study	Data to be collected through registers as well as one-one interviews with HIV positive participants Confidentiality should be kept at all stages of communication Indicator should be disaggregated by gender and age

**Table 10.** Proposed generic outcome indicators for positive attitude.

Objective 1: To encourage positive attitude towards future, health and lifestyle Eligible individuals: participants reached with sport-based HIV/AIDS awareness intervention/reached with HIV/AIDS and health awareness messages during/at sport events, tournaments, training as well as at hubs/centres/sites Participants should at least report positive change in attitude towards one of the three: future, health and lifestyle			
Generic anticipated outcome	Generic outcome indicator	Source	Notes
Positive attitude towards future, health and lifestyle	Percentage of participants reported positive change in attitude towards future, health and lifestyle	This study	Data to be collected through surveys (might be challenging to collect data during mass sport events/tournaments) Indicators should be disaggregated by gender and age

**Table 11.** Proposed outcome indicators for using sport events and tournaments.

Objective 2: To attract participants reached during mass participation at sport events and tournaments to come and participate in small group HIV/AIDS awareness sessions offered at hubs/centres/ sites Eligible individuals: Participants reached with HIV/AIDS and health awareness messages during mass sport events and tournaments			
Generic anticipated outcome	Generic outcome indicator	Source	Notes
Increased percentage of participants reached with small group sport-based HIV/AIDS awareness interventions	Percentage of participants reached during mass sport events and tournaments who came and attended small group HIV/AIDS awareness sessions offered at hubs/centres/sites	This study	Data to be collected through surveys asking a question about where did they hear about the programme, whether they have been reached with HIV/AIDS and health messages during mass events and tournaments offered by specific NGO Indicator should be disaggregated by gender and age

**Table 12.** Proposed outcome indicators for predictors of HIV risk behaviours.

Objective: To reduce HIV risk behaviours			
Generic anticipated outcomes	Generic outcome indicators	Sources	Notes
Delayed sexual debut	Percentage of participants reporting to have had sexual intercourse before the age of 15	Delva et al. (2010) DBE (2012) Shisana et al. (2014) USAID (2013) UNAIDS (2013, 2014, 2015)	Data to be collected through surveys using self-reported HIV risk behaviours questions Pre & post for intervention group If possible have a control group and do pre & post Indicators should be disaggregated by gender and age
Decreased number of multiple sexual concurrent partners	Percentage of participants reporting to have had more than one partner in the past 12 months	Shisana et al. (2014) United Nations, MDG (2008) USAID (2013) UNAIDS (2013, 2014, 2015)	
Increase condom use	Percentage of participants reporting to have had more than one partner in the past 12 months who report the use of condom during their last sexual intercourse	DBE (2012) Shisana et al. (2014) United Nations, MDG (2008) USAID (2013) UNAIDS (2013, 2014, 2015)	
	Percentage of participants reporting to have had more than one partner who report the use of condom the first time they ever had sex, of those whose first sexual encounter happened after being reached with the intervention	Maro et al. (2009) Delva et al. (2010)	
	Percentage of participants reporting to have had more than one partner who report the use of condom during their last sexual intercourse		
Reduction in age-disparate sex/cross-generational sex	Percentage of participants reporting to have had non-marital sex with a partner 10 years older or more than themselves in the last 12 months, of all those who have had non-marital sex in the last 12 months	USAID (2013)

**Table 13.** Continuation of proposed indicators for predictors of HIV risk behaviours.

Objective: To reduce HIV risk behaviours			
Generic anticipated outcomes	Generic outcome indicators	Sources	Notes
Reduction in transactional sex (provision of sex in exchange for material things such as money, airtime, clothing and lunch)	Percentage of participants reporting having transactional sex in the last 12 months	Soul City Institute Health and Development (2010)	Data to be collected through surveys using self-reported HIV risk behaviours questions Pre & post for intervention group If possible have a control group and do pre & post Indicators should be disaggregated by gender and age
Reduction in in alcohol and substance abuse	Percentage of adolescents participants reporting to have had used alcohol or illicit drugs in the past 30 days	Office of Disease Prevention and Health Promotion (2014)	

For both frameworks, the title stating proposed indicators of specific determinants of HIV/AIDS is written. The first row presents objectives, the second row has four columns in this manner: first column: generic anticipated outcomes, second column: proposed generic outcome indicators, third column: sources where the proposed generic outcome indicators were identified/adapted, fourth column notes suggesting data collection methods and disaggregation. For the purpose of the proposed outcome frameworks, please note that participants refer to individuals reached with sport programme/intervention that integrates HIV/AIDS awareness and life skills. These are offered during/at sport events, tournaments and trainings as well as at hubs/centres/sites. Please note that word participants supposedly to appear in the objectives, generic anticipated outcomes, outcome indicators and notes. Due to limited space it is only written on the column presenting proposed outcome indicators. It is important to highlight that where it is written (-this study) as a source, it means proposed outcome indicator was developed by the researcher and suggestions and contribution of the specialists/experts and an official from SRSA were incorporated.

#### 3.6.2. Descriptions of Tables 1–13

A total of 12 objectives, 37 generic anticipated outcomes and 59 generic outcome indicators were proposed from this study and are described below as presented in Tables 1–13. The outcome indicators for sport-based HIV/AIDS awareness programmes are not limited or restricted to the proposed generic outcome indicators presented in Tables 1–13. Other outcome indicators can be further explored to contribute in strengthening monitoring and evaluation of sport-based HIV/AIDS awareness programmes.

Table 1: Improved knowledge of HIV/AIDS: one objective, one generic anticipated outcome and two proposed generic outcome indicators.

Table 2: Raising awareness of HIV risk behaviours. One objective, seven generic anticipated outcomes and ten proposed generic outcome indicators.

Table 3: Raising awareness and access to condoms (male and female condoms): one objective, seven generic anticipated outcomes and ten proposed generic outcome indicators.

Table 4: Uptake of HCT: one objective, three generic anticipated outcomes and six proposed generic outcome indicators.

Table 5: Raising awareness about gender norms that increase risk of HIV infection: one objective, one generic anticipated outcome and seven proposed generic outcome indicators.

Table 6: Uptake of HIV treatment, care and support: one objective, four generic anticipated outcomes and four proposed generic outcome indicators.

Table 7: Uptake of VMMC: one objective, two generic anticipated outcomes and three proposed generic outcome indicators.

Table 8: Use of health services: one objective, one generic anticipated outcome indicators and three proposed generic outcome indicators.

Table 9: Stigma and discrimination: one objective, three generic anticipated outcomes and four proposed generic outcome indicators.

Table 10: Encouraging positive attitude: one objective, one generic anticipated outcome and one proposed generic outcome indicator.

Table 11: Use of sport events and tournaments to attract participants to come and participate in small group HIV/AIDS awareness sessions offered at hubs/centres/ sites: one objective, one generic anticipated outcome and one proposed generic outcome indicator.

Tables 12 and 13: Proposed outcome indicators for predictors of HIV risk behaviours: one objective, six generic anticipated outcomes and eight proposed generic outcome indicators.

The proposed generic outcome indicators can assist the NGOs to improve monitoring and evaluation of their sport-based HIV/AIDS awareness programmes. Furthermore, the use of multiple data collection instruments enables this study to provide options for the use of outcome indicators of sport-based HIV/AIDS awareness programmes of selected NGOs in South Africa. The study can also be a replicable model for other programmes of other NGOs.

## 4. Recommendations

This study has proposed possible generic outcome indicators for sport-based HIV/AIDS awareness programmes. However, a need exists for the improvement of the monitoring and evaluation of sport-based HIV/AIDS awareness programmes by the South African Government as well as NGOs and international stakeholders such as the United Nations. The following recommendations are provided to be considered in order to contribute to the improvement of monitoring and evaluation of sport-based HIV/AIDS awareness programmes. These recommendations are divided into two parts. The first part of recommendations focuses on the role of international organisations, funders, countries, governments and NGOs, followed by recommendations to be considered by selected NGOs examined in this study.

### 4.1. Recommendations for collaborative approach

The United Nations Office on Sport for Development and Peace should provide guidelines on monitoring and evaluation frameworks for sport-based HIV/AIDS awareness programmes. The guidelines on monitoring and evaluation frameworks should be developed in such a manner that they enable the governments of member states and NGOs to adopt the guidelines and contribute to on-going monitoring and evaluation activities at a global level. Funders and governments need to review their funding policies in order to allow an opportunity for donor/government funded NGOs to establish in-house monitoring and evaluation systems, M&E functions and proper information management systems. A need exists for a training module that is specifically tailored to build monitoring and evaluation capacity for NGOs including M&E systems. The training module should be designed and delivered in such a manner that it enables the NGOs to understand the importance of having own in-house monitoring and evaluation systems and functions. The training module should influence the NGOs to recognise monitoring and evaluation as an ongoing activity from the beginning until the end of the programme or project.

In South Africa, a partnership between the following government departments is recommended: Sport and Recreation South Africa, Department of Health and Department of Basic Education should be strengthened to allow the establishment of clear guidelines on how to fast-track the HIV/AIDS activities provided by sport for development NGOs and clubs to ensure the alignment with other health-based organisations. Furthermore, to ensure the alignment with the objectives of the National Strategic Plan for HIV/AIDS, STIs and TB so that sport-based HIV/AIDS awareness programmes can be able contribute in a credible and useful way. Platforms for sharing of information and practices regarding implementation, monitoring and evaluation of HIV/AIDS activities should be encouraged among NGOs and other-health based organisations that use sport as a tool to respond to HIV/AIDS. This will provide an opportunity to learn from one another and to promote best practices in the field of HIV/AIDS.

### 4.2. Recommendations to be considered by selected NGOs

The selected NGOs should establish monitoring and evaluation functions within their organisations. They should take into consideration factors such as organisational roles, responsibilities and capabilities to ensure sustainability of monitoring and evaluation within their organisations. The selected NGOs should develop monitoring and evaluation plans using Theory of change, logic/change models and approaches to improve planning to ensure that anticipated outcomes are realistic and appropriate for M&E systems. It is critical for selected NGOs to ensure the integrity, accuracy and reliability of the output results and data since they are the foundation of achieving the programme’s desired outcomes.

Governments and NGOs should select appropriate study designs and sample sizes when conducting evaluation studies of their sport-based HIV/AIDS awareness programmes. Role players should make use of guidelines from UNAIDS (2015) and Shisana et al. (2014) to identify methods of measurement when using proposed outcome indicators for sport-based HIV/AIDS awareness programmes. This is also necessary to formulate appropriate questions that can be asked during surveys as well as the use of numerators and denominators in order to calculate percentages. The proposed outcome indicators should be disaggregated by age and gender and if possible, by geographical location. The selected NGOs should ensure that their sport-based HIV/AIDS awareness programmes are age appropriate in terms of messages aimed at raising awareness about HIV risk behaviour.

## 5. Conclusion

Although the findings of this study revealed that it will be challenging for selected NGOs to measure directly the contribution of their sport-based HIV/AIDS awareness programmes to the reduction of HIV incidence in South Africa. It can be concluded that the sport-based HIV/AIDS awareness programmes of selected NGOs address similar HIV risk behaviours identified in the objectives of the current National Strategic Plan for HIV, STIs and TB 2012–2016 of South Africa. However, measuring the predictors of HIV risk behaviours directly might be challenging for the selected NGOs considering the challenges such as cost implication and time frame. The overall findings indicated that the proposed outcome indicators for sport-based HIV/AIDS awareness programmes of selected NGOs would rely mostly on measuring change in the knowledge of HIV/AIDS, change in attitude and intention towards HIV risk behaviours. A total of 51 generic outcome indicators were proposed from this study. In addition, eight generic outcome indicators were proposed to measure predictors of HIV risk behaviours. Majority of proposed outcome indicators require the use of rigorous research designs that involve surveys that utilise pre and post questionnaires. If possible the use of control group should be included in the research designs. The selected NGOs can adapt the proposed generic outcomes and indicators based on the settings of their programmes. A collaborative approach by all stakeholders is required, from international organisations, funders, governments, NGOs and communities to strengthening monitoring and evaluation of sport-based HIV/AIDS awareness programmes including other development programmes. This will assist the NGOs that use sport for development to be able to reflect accurately the information about their HIV/AIDS activities and also be able to contribute to on-going monitoring activities at a national and global level as well as to sustainable development.
